# Can Artificial Intelligence be Successful as an Anaesthesiology and Reanimation Resident?

**DOI:** 10.4274/TJAR.2025.251927

**Published:** 2025-12-22

**Authors:** Gökçen Kültüroğlu, Yusuf Özgüner, Savaş Altınsoy, Seyyid Furkan Kına, Ela Erdem Hıdıroğlu, Jülide Ergil

**Affiliations:** 1 University of Health Sciences Türkiye Ankara Etlik City Hospital, Clinic of Anaesthesiology and Reanimation, Ankara, Türkiye

**Keywords:** Anaesthesiology, exam, large language models

## Abstract

**Objective:**

This study aims to compare the performance of artificial intelligence (AI) chatbot ChatGPT with anaesthesiology and reanimation residents at a major hospital in an exam modelled after the European Diploma in Anaesthesiology and Intensive Care Part I.

**Methods:**

The annual training exam for residents was administered electronically. One day prior to this, the same questions were posed to an AI language model. During the analysis, the residents were divided into two groups based on their training duration (less than 24 months: Group J; 24 months or more: Group S). Two books and four guides were used as references in the preparation of a 100-question multiple-choice exam, with each correct answer awarded one point.

**Results:**

The median exam score among all participants was 70 [interquartile range (IQR) 67-73] out of 100. ChatGPT correctly answered 71 questions. Group J had a median exam score of 67 (IQR 65.25-69), while Group S scored 73 (IQR 70-75) (*P* < 0.001). Residents with less than 24 months of training performed significantly worse across all subtopics compared to those with more extensive training (*P* < 0.05). When ranked within the groups, ChatGPT placed eighth in Group J and 47^th^ in Group S.

**Conclusion:**

ChatGPT exhibited a performance comparable to that of a resident in an exam centred on anaesthesiology and critical care. We suggest that by tailoring an AI model like ChatGPT in anaesthesiology and resuscitation, exam performance could be enhanced, paving the way for its development as a valuable tool in medical education.

**Main Points**

• Keeping up with the rapid advancements in medical technologies, pharmaceuticals, and interventions, and integrating them into anaesthesiology education is essential.

• Large language models are artificial intelligence systems trained on huge datasets to understand, analyze, and generate text using deep learning techniques and artificial neural networks.

• Comparing trainee physicians in anaesthesiology and reanimation with artificial intelligence models trained on large datasets, can help guide the design of training programs for anaesthesiology and reanimation residents.

## Introduction

The science of anaesthesiology and critical care is undergoing a significant digital transformation. This makes it necessary to keep up with the rapid developments in medical technologies, drugs and interventions, and to integrate these into education. Combining traditional knowledge and skills training with cutting-edge digital content, including artificial intelligence (AI), holds great potential for enhancing anaesthesia training.^1^

Natural language processing (NLP) is a subfield of AI that focuses on understanding, analyzing, and generating human language, often using large language models (LLMs) for advanced capabilities. The most widely used LLM is ChatGPT^®^ (OpenAI, USA). The latest version, ChatGPT-4, has enhanced its ability to understand and generate human language, and it has been reported to provide more accurate, context-appropriate, and effective responses.^2^

ChatGPT has been included in exams with various content and formats. While its responses were found to be sufficient in some cases, it was observed that improvements were needed in others.^3, 4, 5^ This study aims to test the hypothesis that ChatGPT will outperform anaesthesiology and reanimation residents at a major hospital, in an exam modeled after the European Diploma in Anaesthesiology and Intensive Care (EDAIC) Part I.

## Methods

### Ethical Statement

The Scientific Research Evaluation and Ethics Board of University of Health Sciences Türkiye, Ankara Etlik City Hospital determined that this singlecentre, cross-sectional study did not require ethical approval (date: 12/06/2024, approval no.: AESHBADEK-2024-546). All participants were informed about the study, and their written consent was obtained.

When writing this article, an AI program was used to correct spelling and grammar (https://chat.openai.com).

### Study Design

The study was conducted in the Clinic of Anaesthesiology and Reanimation at University of Health Sciences Türkiye, Ankara Etlik City Hospital. The exam was a standard part of the annual resident training programme, and all residents were informed about its schedule, content and format in advance. However, residents were kept blind to the protocol until informed about the study just before the exam.The study aimed to include all residents trained in the department. Accordingly, the exam date (14 October 2024) and time (9:00 AM, UTC+3) were determined. Residents gathered in the meeting rooms 15 minutes before the exam, and the exam was conducted simultaneously in six different rooms, each overseen by a different proctor. Participants were instructed to log in to the exam via their mobile phones and complete it within the allotted time. Once the exam period expired, the exam was automatically submitted online. At the end of the exam, the questions and answers were shared with all participants. The results were evaluated using e-forms. In the analysis of the results, residents were divided into two groups based on their training duration: those with less than 24 months (Group J) and those with 24 months or more (Group S).

One day before the questions were administered to the participants, they were directed to the ChatGPT application by two consultants (S.F.K., Y.Ö.) in anaesthesia and critical care, who were blind to the study protocol. A new user profile was created to prevent bias. All information and questions were presented in Turkish. Before starting the exam, the question “Which ChatGPT model are we using?” was asked, and it was confirmed that the ChatGPT-4 model was used. Then, the following information was provided to ChatGPT: “You are an anaesthesiology and reanimation resident working in a large hospital”. I have prepared an evaluation exam for you. The purpose of the exam is to compare your knowledge with that of anaesthesiology and critical care resident physicians working in a large hospital. The exam topics are as follows: Anaesthesia equipment and monitors, clinical pharmacology, anaesthesia management, regional anaesthesia and pain, and intensive care. The exam consists of 25 questions, with five questions for each topic. Each question has four options. You must answer each option as “True” or “False”. The questions will be asked of you one by one. I would like you to complete this exam within 30 minutes. The programme indicated that it was ready to perform its functions after receiving this information. The questions were copied from a word processor document into ChatGPT’s chat box for the answers. The first generated answer was taken as the final response, and the option to regenerate the answer was unavailable. The answers were marked on an optical form by the assigned physicians. A total of 100 responses were obtained, with each correct answer scored as one point. There was no penalty for incorrect answers or unanswered questions.

### Preparing the Exam

The exam was prepared by two physicians (G.K. and Y.Ö.) who have been specialists in anaesthesiology and resuscitation for at least 10 years. The questions were designed in a manner similar to the EDAIC Part I exam questions. Five main topics were identified: “Anaesthesia equipment and monitors, Clinical pharmacology, Anaesthesia management, Regional anaesthesia and pain, and Intensive care”. A total of 25 questions were created, with five questions from each topic. Each question included four statements which were labelled as either “True” or “False”. Two primary textbooks were used as references for preparing the questions.^6, 7^ For topics such as sepsis, acute respiratory distress syndrome, cardiopulmonary resuscitation, and nutrition, the most recent guidelines adopted by our clinic were utilised.^8, 9, 10^ An associate professor (S.A.) and a professor (J.E.), who were blinded to the study protocol, reviewed the questions for accuracy and validity. After the ChatGPT exam was completed, the questions were converted into an online form for physicians to answer. The questions did not include tables or figures.

### Outcome Measures

The primary outcome of the study was the comparison of exam performance among anaesthesiology and reanimation residents and the ChatGPT programme, measured by the total number of correct answers. Secondary outcomes included the relationship between residents’ training duration and their exam performance, ChatGPT’s performance compared to residents with varying levels of training, and the concordance between ChatGPT and the reference in answering the exam questions.

### Statistical Analysis

All statistical analyses were performed using IBM SPSS statistics (version 25.0, IBM Corp., Armonk, NY, USA). Descriptive statistical methods [frequency, percentage, median and interquartile range (IQR 25-75)] were used to evaluate the study data. The normality of the data distribution was assessed using the Shapiro-Wilk test. Non-parametric tests were preferred for the analysis of data that did not follow a normal distribution. The Mann-Whitney U test was employed to compare the two groups. The level of agreement between the responses provided by ChatGPT and the reference answers was assessed using Cohen’s kappa coefficient. The Kappa values were interpreted based on the classification proposed by Landis and Koch: <0, poor agreement; 0-0.20, slight agreement; 0.21-0.40, fair agreement; 0.41-0.60, moderate agreement; 0.61-0.80, substantial agreement; and 0.81-1.00, almost perfect agreement.^11^ Additionally, McNemar’s test was performed to determine whether there was a statistically significant difference between ChatGPT’s correct and incorrect responses compared to the reference answers. A significance level of *P* < 0.05 was considered for all analyses.

## Results

**Figure 1:**
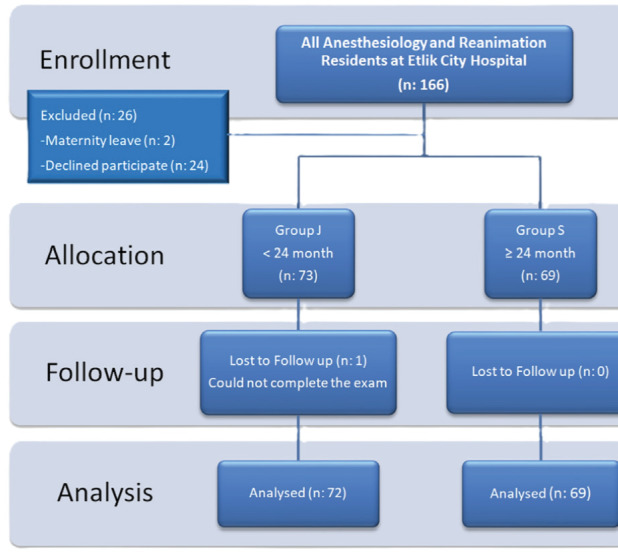
Flowchart of the study

A total of 166 residents worked in the Clinic of Anaesthesiology and Reanimation at University of Health Sciences Türkiye, Ankara Etlik City Hospital. Two residents were unable to participate in the examination as they were on maternity leave, and 24 residents did not consent to the use of their exam results for research purposes. Additionally, one resident did not complete the exam within the required timeframe. Consequently, the exam results of 141 residents were included in the study (Figure 1).

ChatGPT answered all the options of the 25 questions (100 answers) and completed the exam in approximately 18 minutes. Only for the first question did it provide explanations alongside the “true/false” answers. For the other questions, it only provided “true/false” answers. It did not leave any questions unanswered. At the end of the exam, it did not wish to change any of its answers.

**Figure 2:**
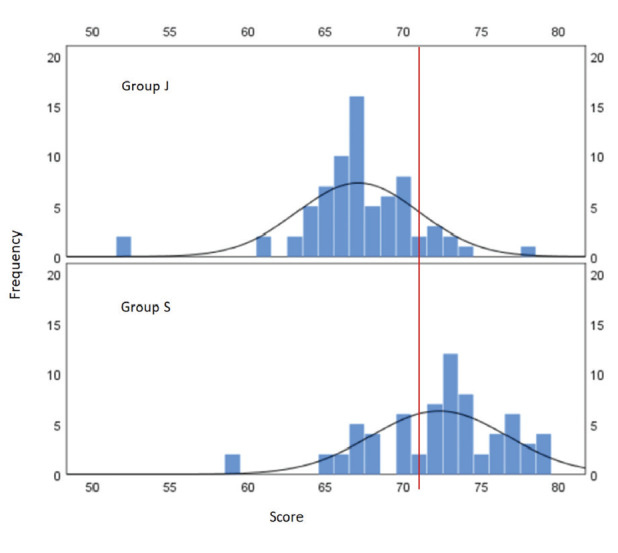
The exam performance of the groups. ChatGPT’s score marked by a red line.

The median exam score for all participants was 70 out of 100 (IQR 67-73). ChatGPT’s correct answer count was found to be 71 (Table 1). Residents with less than 24 months of training had significantly different exam results, both overall and in all subtopics, compared to those with longer training (Table 1). When ranked by exam results, ChatGPT placed 54^th^ in this sample. The exam performance of the groups is presented in Figure 2, with ChatGPT’s score marked by a red line. When the exam results were ranked by group, ChatGPT ranked eighth in Group J. In the ranking within Group S, ChatGPT ranked 47^th^.

ChatGPT provided correct answers to all options in five out of 25 questions. There were no questions for which all answers were incorrect. However, for two questions, the majority of options (3/4) were answered incorrectly. The first of these questions was related to the anatomy of peripheral nerve blocks, where incorrect answers were given for options related to the anatomical structures of the obturator, femoral, and axillary nerves. The second question was related to resuscitation in a hypothermic patient, where incorrect answers were provided for compression, adrenaline administration, and defibrillation. The highest number of correct answers was found in the “Anaesthesia Management” section (17/20 points). The sections with the most incorrect answers were “Clinical Pharmacology, Regional Anaesthesia-Pain and Intensive Care” (13/20 points for each).

The responses given by the ChatGPT-4 language model were compared with the reference answers in Table 2. Cohen’s Kappa value was calculated as κ=0.38 (*P*=0.000). The significance of McNemar’s test was found to be *P*=0.137.

## Discussion

It is important for physicians and residents working in medicine to recognize NLP models like ChatGPT-4, evaluate their applicability, and examine their limitations.^12^ In response to the question, “Is ChatGPT a successful resident?”, ChatGPT scored 71 points in a 25-question, 100-point exam similar to the EDAIC Part I, and this score was very close to the median score for all residents. When the residents were divided into two groups based on their training duration, it was observed that ChatGPT provided more correct answers than most of those with less than 24 months of training. However, ChatGPT scored lower than most of those with longer training. ChatGPT’s responses to the exam showed a moderate level of agreement with the reference answers and did not reveal any significant differences.

ChatGPT has been reported to achieve an accuracy rate of 65-75% on the American Heart Association’s Basic Life Support (BLS) and Advanced Cardiovascular Life Support (ACLS) exams. In this study, the authors utilised scenario-based and single-answer questions from the 2016 BLS and ACLS examinations. While the correct answers provided by ChatGPT did not meet the passing threshold of 84%, the results showed a significantly better alignment with resuscitation guidelines compared to previous studies.^13^ In another study, ChatGPT demonstrated an accuracy rate of approximately 80% on the 2022 and 2023 National Medical Licensing Examination in Japan, meeting the passing thresholds for these exams.^14^ Similarly, in a study evaluating the performance of ChatGPT on an e-Fellowship of the Royal College of Anaesthetists (FRCA) primary exam, it achieved approximately 70% accuracy in multiple choice questions (MCQs). Our results show that ChatGPT can answer Primary FRCA MCQ practice questions at a level close to the 2019 exam pass mark, which was 0.713.^15^ However, ChatGPT-4’s performance on the Japan Society of Anaesthesiologists (JSA)-certified anaesthesiologist exams was limited, with success rates of 51% and 49% observed for the 2021 and 2022 examinations, respectively.^16^ In another study aimed at evaluating ChatGPT’s level of anaesthesiology expertise using questions styled after the American Board of Anaesthesiology’s (ABA) written examinations, the model achieved a moderate success rate of 56%.^17^ In our study, ChatGPT-4 demonstrated a noteworthy performance rate of 71%. This success rate represents a promising indication of its potential to serve as a reliable resource for passing actual board exams or maintaining certification standards.

It could be anticipated that the participants, divided into two groups based on their years of training, would show differences in the subtopics and in their total scores. It was an expected outcome that individuals with more time spent in professional training would achieve superior performance. However, it was surprising to observe that AI lagged behind many of the senior residents. In a study conducted in medical biochemistry, ChatGPT’s performance was compared with that of 100 medical students. The exam included multiple-choice and subjective questions, and it was found that ChatGPT performed better than in the students’ responses.^18^ In another study, the “Progress Test Medicine” in Germany was administered to ChatGPT. In this multiple-choice exam, ChatGPT outperformed almost all German medical students in the first to third years of a six-year medical programme.^19^ In another study, it was noted that ChatGPT achieved better results than both medical students and residents in a written neurosurgery exam consisting of board-like questions.^20^ In a study involving histopathological examinations, ChatGPT fell behind pathology residents in all responses.^21^ The success of ChatGPT in this study was very close to the performance level of all residents. The fact that the exams were multiple-choice, that they involved images in pathological and radiological evaluations, and that some exams were open-ended and required reasoning will certainly affect the differences in these results. In this study, the exam questions required both knowledge and reasoning. No questions containing images or tables were asked, and there were no open-ended questions.

The agreement between ChatGPT and the reference answers was moderate (κ=0.38), with no significant differences observed between them. This outcome may reflect not only the AI algorithm but also the methodology used in preparing the questions. The questions were developed based on both fundamental textbook knowledge and updated guidelines. The answers were not designed to allow for open interpretation by ChatGPT: they were limited to two options: true/false. Before the questions were shared electronically with participants, they were tested using ChatGPT to check for any prior exposure by the AI. In some studies, evaluations of ChatGPT are carried out using questions from previously administered exams; however, this approach increases the risk of bias.^22^ Even a single instance of questions being entered into electronic systems-whether for exam preparation, distribution, printing, or as a result of individuals searching for them online-can familiarize AI applications with these questions, potentially leading to artificially inflated performance scores. In our study, particular care was taken to address this issue, and the questions were saved only in a word processor document and not uploaded to any electronic platform. Another factor that likely influenced the alignment between ChatGPT’s responses and the reference answers is the language used. Although this program, developed as a LLM, has a translation feature in many languages, the fact that the exam was conducted in Turkish may have affected the program’s performance and its alignment with the reference answers.

This study did not involve open-ended questions-only two options, true or false, were provided. Even under the conditions we set, ChatGPT could not express uncertainty and never responded with “I don’t know” or “I don’t want to answer”. In some studies, ChatGPT has been observed to justify incorrect answers as convincingly as correct ones, a behaviour that is not uncommon in LLMs, sometimes referred to as "hallucination".^19^ The question of whether integrating ChatGPT into medical education programs would be beneficial is at the core of this and similar studies. However, its inability to express uncertainty and its tendency to misinterpret information limit its usefulness in medical education.

### Study Limitations

The potential benefits of the study include gaining insights into ChatGPT’s proficiency in anaesthesiology and critical care topics and generating data to inform future adjustments to the assistant training curriculum related to AI. However, the study had limitations; it only included residents trained in a single clinic, and generalizing the findings may be problematic due to the homogeneous training background of the participants. Although the questions were carefully designed to cover all relevant topics, they may not have encompassed every aspect of anaesthesia, analgesia and critical care procedures. Additionally, the language used in ChatGPT was set to Turkish.

## Conclusion

ChatGPT demonstrated an average performance at the level of a resident in an exam focused on anaesthesiology and critical care. While it may provide guidance for beginner-level residents, it generated inadequate responses compared to more experienced residents. By training an AI like ChatGPT in anaesthesiology and resuscitation, it could demonstrate higher exam performance and be developed into an AI that can be utilized in anaesthesiology and reanimation education.

## Ethics

**Ethics Committee Approval:** The Scientific Research Evaluation and Ethics Board of University of Health Sciences Türkiye, Ankara Etlik City Hospital determined that this singlecentre, cross-sectional study did not require ethical approval (date: 12/06/2024, approval no.: AESHBADEK-2024-546).

**Informed Consent:** All participants were informed about the study, and their written consent was obtained.

## Figures and Tables

**Table 1. Correct Answers of Anaesthesiology and Reanimation Residents and ChatGPT:** 

-	**Total** **n=141 Median** **(IQR 25-75)**	**Group J** **(<24 month)** **n=72 Median** **(IQR 25-75)**	**Group S** **(≥24 month)** **n=69 Median** **(IQR 25-75)**	P*	**ChatGPT**
Anaesthesia equipment and monitors	15 (14-16)	14.5 (14-15)	16 (15-17)	<0.001	15
Clinical pharmacology	12 (11-13)	11 (11-12)	12 (11-14)	0.002	13
Anaesthesia management	16 (15-18)	16 (14-16)	17 (16-18)	<0.001	17
Regional anaesthesia and pain	15 (13-16)	14 (13-15)	15 (14-16)	0.007	13
Intensive care	12 (11-13)	11 (10.25-12)	13 (11-14)	0.001	13
Total score	70 (67-73)	67 (65.25-69)	73 (70-75)	<0.001	71

*It is comparison of Group J and Group S

IQR, interquartile range.

**Table 2. Comparison of ChatGPT and Reference Answers:** 

-		**Reference**		**Total**
		**True**	**False**	
ChatGPT	True	49	19	68
	False	10	22	32
Total		59	41	100
